# An Immunologic Compatibility Testing Was Not Useful for Donor Selection in Fecal Microbiota Transplantation for Ulcerative Colitis

**DOI:** 10.3389/fimmu.2021.683387

**Published:** 2021-06-04

**Authors:** Manuel Ponce-Alonso, Carlota García-Hoz, Ana Halperin, Javier Nuño, Pilar Nicolás, Adolfo Martínez-Pérez, Juan Ocaña, Juan Carlos García-Pérez, Antonio Guerrero, Antonio López-Sanromán, Rafael Cantón, Garbiñe Roy, Rosa del Campo

**Affiliations:** ^1^ Servicio de Microbiología, Hospital Universitario Ramón y Cajal and Instituto Ramón y Cajal de Investigación Sanitaria, Madrid, Spain; ^2^ Red Española de Investigación en Patología Infecciosa (REIPI), Madrid, Spain; ^3^ Servicio de Inmunología, Hospital Universitario Ramón y Cajal and Instituto Ramón y Cajal de Investigación Sanitaria, Madrid, Spain; ^4^ Servicio de Cirugía General y del Aparato Digestivo, Hospital Universitario Ramón y Cajal, Madrid, Spain; ^5^ Organización Nacional de Trasplantes, Madrid, Spain; ^6^ Servicio de Gastroenterología y Hepatología, Hospital Universitario Ramón y Cajal, Madrid, Spain; ^7^ Universidad Alfonso X El Sabio, Madrid, Spain

**Keywords:** mucosal immunity, fecal microbiota transplantation, ulcerative colitis, donor selection, immunological compatibility

## Abstract

Fecal microbiota transplantation (FMT) is an effective procedure against *Clostridioides difficile* infection (CDI), with promising but still suboptimal performance in other diseases, such as ulcerative colitis (UC). The recipient’s mucosal immune response against the donor’s microbiota could be relevant factor in the effectiveness of FMT. Our aim was to design and validate an individualized immune-based test to optimize the fecal donor selection for FMT. First, we performed an *in vitro* validation of the test by co-culturing lymphocytes obtained from the small intestine mucosa of organ donor cadavers (n=7) and microbe-associated molecular patterns (MAMPs) obtained from the feces of 19 healthy donors. The inflammatory response was determined by interleukin supernatant quantification using the Cytometric Bead Array kit (B&D). We then conducted a clinical pilot study with 4 patients with UC using immunocompetent cells extracted from rectal biopsies and MAMPs from 3 donor candidates. We employed the test results to guide donor selection for FMT, which was performed by colonoscopy followed by 4 booster instillations by enema in the following month. The microbiome engraftment was assessed by 16S rDNA massive sequencing in feces, and the patients were clinically followed-up for 16 weeks. The results demonstrated that IL-6, IL-8, and IL-1ß were the most variable markers, although we observed a general tolerance to the microbial insults. Clinical and colonoscopy remission of the patients with UC was not achieved after 16 weeks, although FMT provoked enrichment of the *Bacteroidota* phylum and *Prevotella* genus, with a decrease in the *Actinobacteriota* phylum and *Agathobacter* genus. The most relevant result was the lack of *Akkermansia* engraftment in UC. In summary, the clinical success of FMT in patients with UC appears not to be influenced by donor selection based on the explored recipient’s local immunological response to FMT, suggesting that this approach would not be valid for FMT fecal donor optimization in such patients.

## Introduction

The interaction between the gut microbiota and immune system plays a relevant role in the etiopathogenesis of inflammatory bowel diseases, such as ulcerative colitis (UC) (1, 2). A loss of immune tolerance to bacterial luminal antigens has been reported, triggering a local response (activation of dendritic cells), which spreads regionally and results in T-cell differentiation toward T helper (TH)-1, TH-2, TH-17 or regulatory T (Treg) phenotypes that ends in a “storm” of proinflammatory cytokines and chemokines, generating and perpetuating tissue damage (3). In fact, relevant differences in microbiota composition between inflamed and noninflamed intestinal mucosa have been reported in UC, according with the activity status of the disease (2, 4). The genetic basis of UC indicates the protective role of various human polymorphisms in the interleukin (IL)-27 gene (5), as well as the predominant involvement of a TH-17 response, in close relationship with the intestinal microbiota (6, 7).

The therapeutic approach for UC is currently based on inflammation control. Fecal microbiota transplantation (FMT) has emerged as a therapeutic option for UC, aiming to modulate the gut microbiota composition and thus the chronic inflammatory status (8, 9), although its clinical effectiveness is not as high as reported for *Clostridioides difficile* infection (CDI) (10, 11). Significant efforts has been made in recent years to determine the reasons behind this suboptimal performance, and a number of authors have suggested the importance of the donor’s microbial composition as a determinant for FMT success in UC (12, 13), particularly in relation to ecosystem diversity (14) or the so-called super-donor phenomenon (15). A recent randomized controlled trial identified specific taxa from donor feces associated with higher clinical response rates in patients with UC who underwent FMT (16). Moreover, fecal mixing from various donors has been tested for higher FMT success rates (17). Despite this, donor selection for FMT in UC is not specific, resulting in random matching in most cases, and the molecular mechanisms by which the autoimmune response is modulated have not yet been elucidated.

The mucosal immune system surrounding the digestive tract protects against the entry of potential pathogens and simultaneously remains tolerant to the resident microbiota (18). Nevertheless, the distinction between commensal and pathogenic bacteria is usually hard to establish and often depends on concrete lineage-specific but species-independent virulence factors. One of the aims of FMT in UC is to modulate the dialogue between the gut microbiota and the mucosal immune system, although several mechanisms could be implicated. In that sense, the success could be determined by the interaction of the two players once the implantation of the donor microorganisms has been completed. In this study, we designed a test based on the recipient’s mucosal immune response against the donor’s microbiome to optimize the fecal donor selection in FMT. First, we evaluated and optimized the viability and performance of the test using immunocompetent cells from jejunal specimens obtained from 7 cadaver organ donors, which were exposed to the microbe-associated molecular patterns (MAMPs) of 19 fecal samples from unrelated healthy donors. We then validated the test’s clinical utility in 4 patients with UC who underwent FMT, employing the compatibility test results to guide the donor selection by confronting immune cells obtained from rectal biopsies against the MAMPs of 3 potential fecal donors. Lastly, we evaluated the impact of FMT in the gut bacterial ecosystem of these patients with UC and the intestinal microbiome of the transplant patients with CDI who shared the same fecal donors.

## Materials and Methods

### *In Vitro* Optimization of the Compatibility Test

The test is based on exposing immunocompetent cells from either the recipient’s gut mucosa or peripheral blood to antigenic MAMPs determinants of the gut of potential donors, determining cytokine production and selecting the donor with the least inflammatory reaction.

Test optimization was performed using immunocompetent cells recovered from jejunal segments and peripheral blood mononuclear cells extracted from 7 cadaver organ donors that were maintained alive through perfusion. Our center’s ethics committee (Reference 253/17) approved the use of the cadavers, with the inclusion criteria of the donor family’s acceptance and the complete integrity of the digestive system, while the exclusion criterion was a history of intestinal tumors or systemic infectious diseases. During the surgery, a fecal aliquot from the intestinal lumen was collected as a representative sample of the cadaver’s microbiota.

Immunocompetent mucosal cells were recovered from a 3-cm jejunal segment removed during the organ donation surgery. After resection, the jejunum segment was submerged in Roswell Park Memorial Institute (RPMI) 1640 Complete Medium (CM) (Life Technologies/Gibco, 11875-085) supplemented with 10% fetal bovine serum (Life Technologies/Gibco 16140-063), imipenem, gentamicin, vancomycin, and fluconazole, all at a concentration of 100 mg/l to inhibit the growth of the autochthonous microbiota. We manually stripped the mucosal layer and incubated the obtained fragments in 50 ml of RPMI 1640 CM supplemented with 10% fetal bovine serum (Life Technologies/Gibco 16140-063), 50 U/ml collagenase (Sigma-Aldrich GE17-1440-02), and 100 U/ml DNAse I recombinant grade I (Sigma-Aldrich 0436282001) for 120 min at 37°C with continuous stirring. The liberated cell suspension (intraepithelial lymphocytes, lamina propria leukocytes, and epithelial cells) was run up and down through an 18G needle and filtered through a cell strainer to remove debris. Cells were pelleted, counted, and suspended in RPMI CM at a density of 200,000 leukocytes/ml. We labeled 100 µl of the cell suspension with anti-CD45 monoclonal antibodies (Becton Dickinson) and fully acquired it in a flow cytometer (FACSCanto II, Becton Dickinson), counting the gated cells. Prior to the donor’s death, 5 ml of peripheral blood was obtained, and the mononuclear cell fraction was purified by Ficoll-Paque density gradient (Sigma-Aldrich, GE17-14440-02).

We employed fecal aliquots from 19 unrelated healthy donors of our *C. difficile* FMT program as the antigenic stimulus. Feces were collected and maintained at −80°C until use. To obtain MAMPs determinants, 0.5 g of feces were completely solubilized in 5 ml of water, boiled for 15 min, centrifuged at 1500 rpm for 5 min, only saving the supernatant.

Co-cultures of the immunocompetent cells with alive bacteria were unsuccessful by premature apoptosis of the cells, and consequently we decide to use MAMPs in a cell culture plate (Thermo Fisher Scientific, USA) at 37 °C and 5% carbon dioxide. The immunological response of the immunocompetent cells was determined by measuring the concentration of inflammatory interleukins (IL-1β, IL-6, IL-8, IL-10, and IL-12p70) and tumor necrosis factor alpha (TNFα) using the Human Inflammatory Cytokine Cytometric Bead Array kit (Becton Dickinson, USA) in a flow cytometer.

We first assayed various settings for the dilution of MAMPs (1, 1/100, and 1/1000) and the incubation period (6, 18, and 24 h) to define the optimal conditions that preserve cell viability during cultivation and ensure the recovery of the cytokine signal. To this end, we employed the cells extracted from the jejunal segment of the first recruited organ donor and its own microbiota as an antigen stimulus. In addition, we tested if the extraction method conserved the integrity of the response capability of the intestinal immunocompetent cells. To do so, we measured the intracellular production of IFNγ and TNFα from immunocompetent CD45+ intestinal cells under a PMA-ionomycin stimulus and 3 different fecal microbiome extractions.

After setting the optimal conditions of the test, we examined whether the inflammatory response of immunocompetent gut mucosal cells against fecal MAMPs was comparable to that of peripheral cells in order to validate our compatibility test using a noninvasive sample (peripheral blood). To this end, we cultivated the jejunal and peripheral cells recovered from the 7 aforementioned cadavers (recipients) with MAMPs from feces of 19 putative donors and their own fecal microbiota. We included a negative control without antigen stimulus in each experiment.

### *In Vivo* Validation in UC Patients

We recruited 4 patients with UC under the following inclusion criteria: extensive moderate UC refractory to conventional drug therapy and consent to be included in the study and to undergo FMT. Rectal biopsies including mucosal-associated lymphoid tissue were obtained from the enrolled patients. Immunocompetent mucosal cells were liberated by enzymatic digestion after introducing the biopsies into 5 ml of RPMI 1640 CM supplemented with collagenase (50 U/ml) and DNA-ase (100 U/ml), incubated under vigorous stirring for at least 2 h at 37°C, with occasional homogenization by repeated needle aspiration. The mix was centrifuged at 1,800 rpm for 7 min, and the resulting pellet (containing intraepithelial lymphocytes, lamina propria leukocytes and epithelial cells) was suspended in 1 ml of fresh CM supplemented with imipenem, gentamicin, vancomycin, and fluconazole (100 mg/l each). We estimated the cell concentration by anti-CD45 labeling and adjusted it to 200,000 CD45+ leukocytes/ml.

For each patient with UC, the fecal microbiota from 3 unrelated donors previously recruited for our FMT program for CDI were processed, and MAMPs co-cultures of immunocompetent mucosal cells were performed as described above, reserving a culture of immunocompetent cells as a negative control to estimate the baseline cytokine production. After incubation, the entire well content was transferred to Eppendorf tubes and centrifuged at 14,000 rpm, collecting the supernatants that were stored at −80°C until cytokine determination. Lastly, cytokine determination was performed in a flow cytometer, as previously explained. The candidate whose fecal MAMPs triggered the mildest response in each patient was selected as the fecal donor.

### FMT Procedure and Bacterial Engraftment Evaluation

We transferred the donor’s microbiota to the patients with UC by colonoscopy using 100 g of fresh donor feces (less than 6 hours and conserved in anaerobiosis) previously suspended in 500 ml of distilled water and filtered to eliminate fiber and other solid residues. Additionally, patients were provided of 4 frozen individual syringes of 50 ml each with a similar fecal solution that kept at home at -20°C until self-administration by enema one per week (in total 4 instillations obtained from another 100 gr of feces). We assessed the engraftment of the transferred bacteria in fecal samples collected before and after 2 weeks, 1 month, and 2 months of FMT. We also included in the analysis 4 fecal samples from 2 patients with CDI (pre-FMT and post-FMT) who underwent FMT by a single colonoscopy using the same fecal donors as those for UC, to compare the FMT engraftment characteristics of the two diseases.

We determined the bacterial composition of the fecal samples by polymerase chain reaction, amplification of the 16S rDNA V3-V4 region, and massive sequencing (2x300 bp) on a MiSeq (Illumina, San Diego, CA, USA) platform. We analyzed the raw sequencing data using the QIIME 2 bioinformatics platform 2020.8 distribution (19), which were deposited in Genbank (PRJNA702025) (for accession details, see **Supplementary Data**). In brief, FASTQ files were demultiplexed and quality assessed using the q2-demux plugin. We then performed denoising, filtering, and chimera removal with the DADA2 pipeline (via q2-dada2 plugin), thereby identifying all amplicon sequence variants (ASVs) and their relative abundance in each sample. To minimize the number of spurious ASVs, those unique sequences with a total abundance lower than 7 reads across all samples were filtered out. ASVs were taxonomically classified by using the classify-sklearn naïve Bayes taxonomy classifier (via the q2-feature-classifier plugin) against the SILVA 138 database (20). We performed a diversity analysis using the q2-diversity plugin, after samples were normalized *via* rarefaction (subsampled without replacement). The diversity analysis comprised alpha diversity metrics (Chao1, Shannon index, and Faith-PD, which measure the degree of microbiome diversity) and beta diversity metrics (unweighted UniFrac, which measures differences in microbiome composition between groups of samples). All statistical tests were conducted *via* the q2-diversity plugin.

### Clinical Follow-Up of UC Patients

UC patients were followed-up for 16 weeks after FMT. Clinical UC activity was assessed by using the Simple Clinical Colitis Activity Index (SCCAI), and the Mayo endoscopic score. In addition, biologic markers (fecal calprotectin) and adverse events were recorded, and a colonoscopy examination was performed at the end of the follow-up.

## Results

### *In Vitro* Optimization of the Test

**Figure 1:**
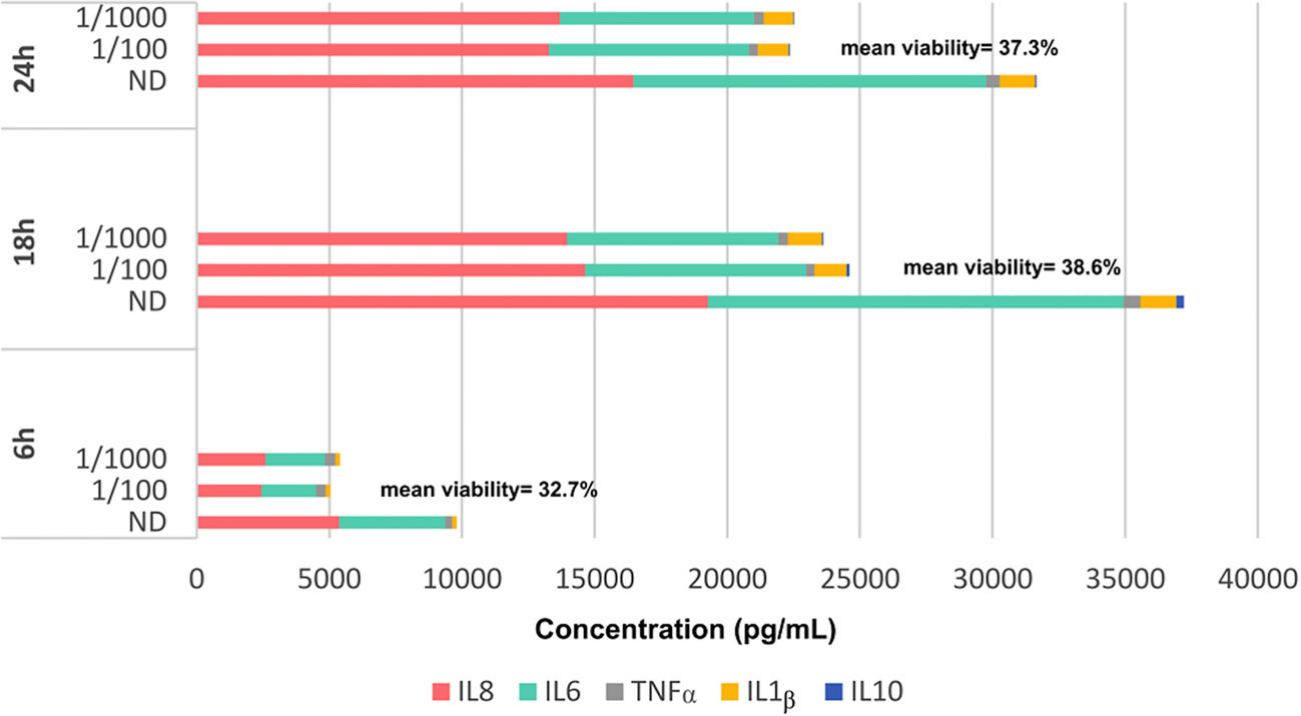
Cytokine production of lymphoid cells obtained from a peripheral blood sample of a healthy volunteer under different incubation times (6, 18 and 24 h) and different dilutions of the fecal supernatant [non-diluted (ND), 1/100 and 1/1000]. Mean cell viability measured at each time is also showed.

As co-cultures with alive bacteria provoked apoptosis of the immunocompetent cells, the most suitable conditions for ensuring their viability corresponded to the use of the non-diluted MAMPs supernatant and incubations of 18 h (**Figure 1**). These conditions were set as the standard for the following experiments. In addition, the extraction technique preserved the integrity of the intracellular immune response capability of the intestinal cells, as shown in supplementary material (**Figure S1**).

**Figure 2:**
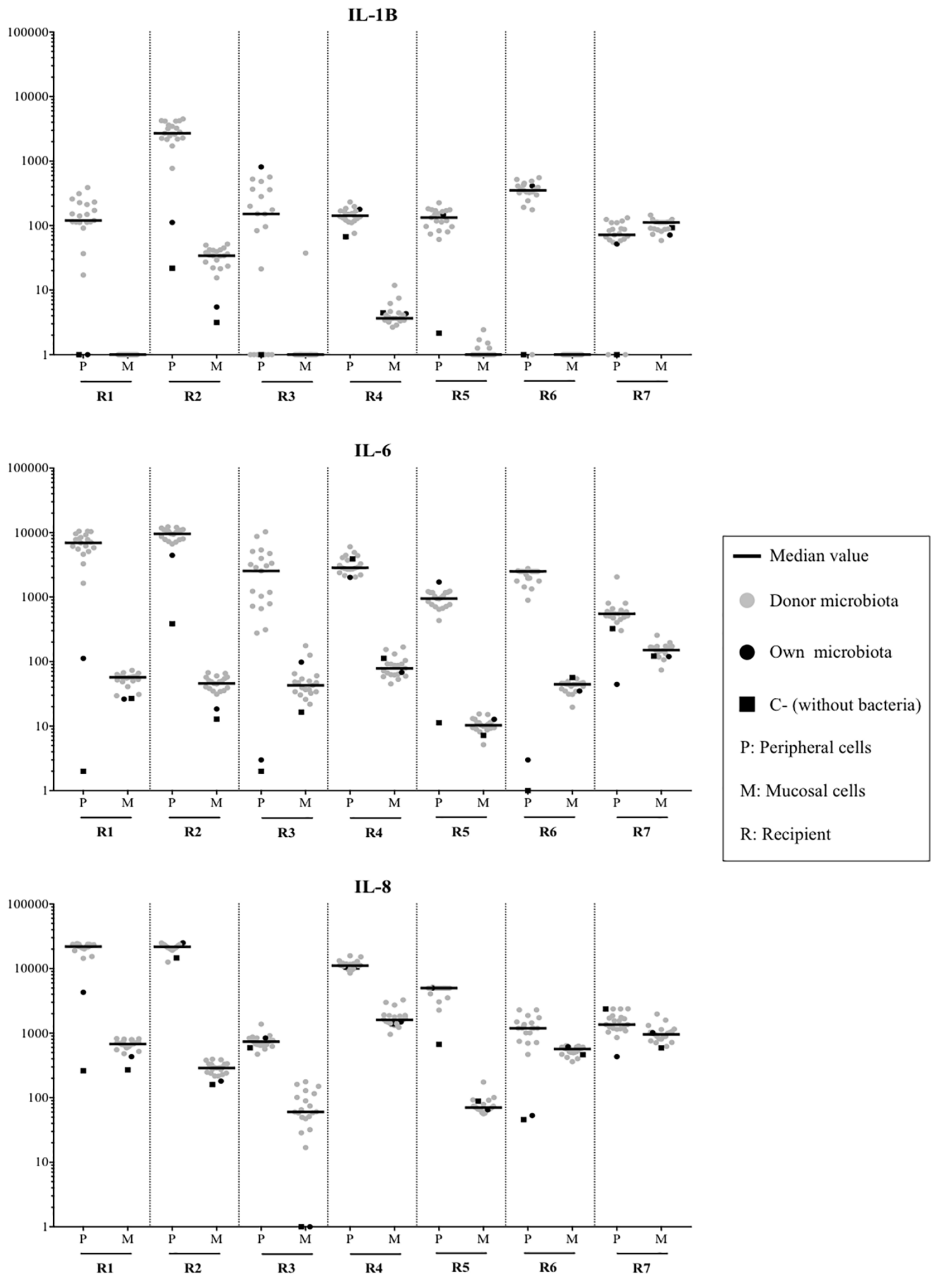
Comparison of peripheral (P) and mucosal (M) immunological response for microbiota from 19 healthy donors, their own microbiota and a negative control without antigenic stimulus. The most variable markers (IL-1β, IL-6 and IL-8) were shown. To note the logarithm scale of the Y axis.

**Figure 3:**
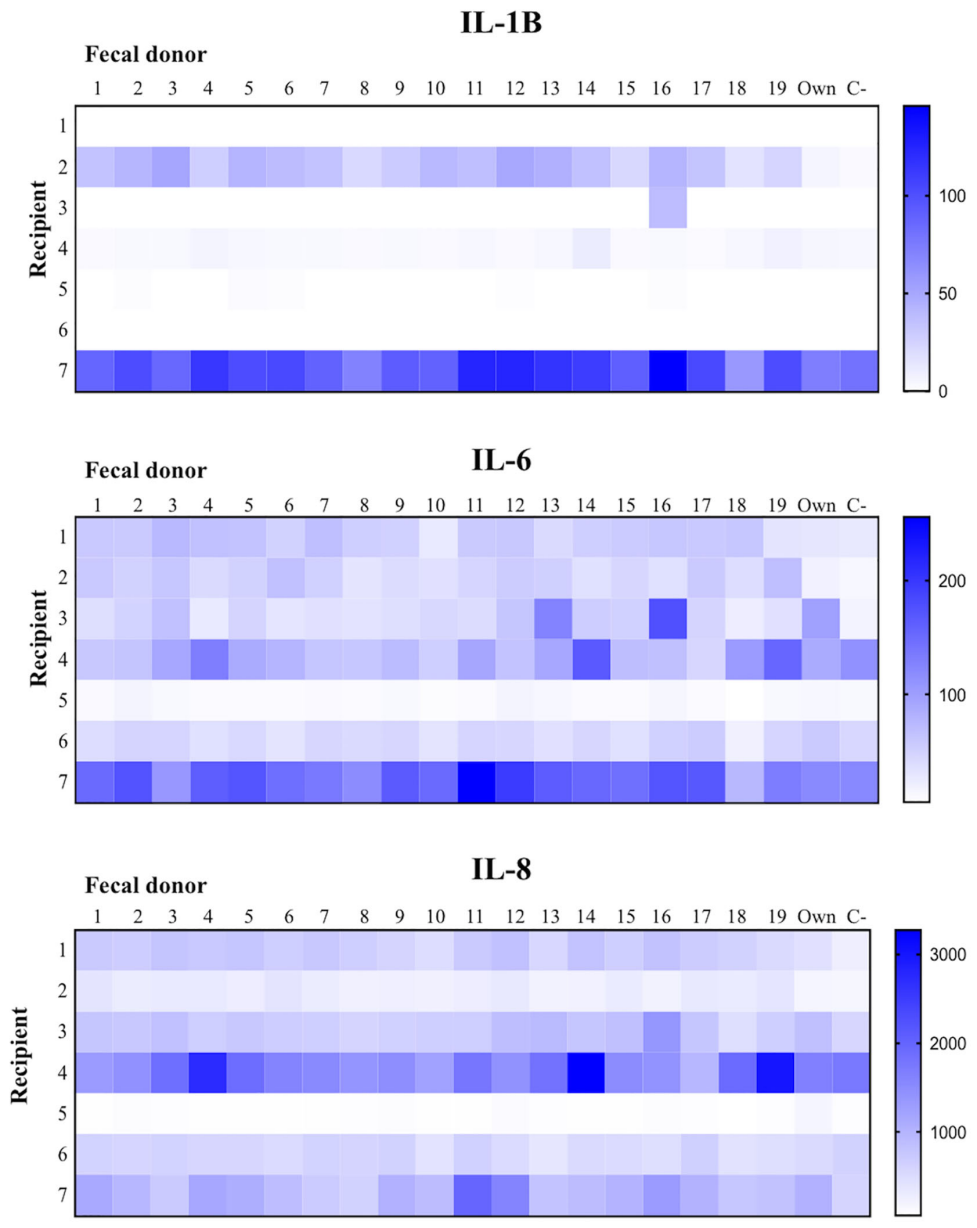
Intrarecipient variability immunological activation (only with mucosal associated immunocompetent cells) against microbiota from 19 healthy donors, their own microbiota and the negative control without microorganisms.

**Figures 1** and **2** illustrate the immunological response of the mononuclear cells obtained from the 7 cadavers against the 20 different fecal MAMPs supernatants. The cytokine production pattern of the jejunal mucosal cells was not comparable to that of the peripheral blood mononuclear cells (**Figure 2**), ruling out the possibility of using this noninvasive sample as an alternative to intestinal biopsy. Mucosal cells showed a high variability of IL-8, IL-6, and IL-1β production, whereas the concentration of TNFα, IL-10, and IL-12 were mostly undetectable in all experiments (**Figure 3**). Cadaveric organ donors 4 and 7 showed the widest range of mucosal cytokine response, while cadaveric organ donor 6 generated the most homogeneous response against all tested MAMPs: 19 from possible donors and their own bacterial ecosystem.

Although we observed considerable variability in the cytokine profiles, it is important to note that these levels were not particularly high, especially if we compare them with those produced by peripheral blood cells. Interestingly, the fecal microbial determinants from donor 18 generated the lowest mucosal immune response in 4 cadavers (numbers 3, 5, 6, and 7); in contrast, donor 18 provoked the third highest cytokine production in recipient 4. The least proinflammatory response from the two remaining cadavers (1 and 2) was generated by their own MAMPs. None of the 19 MAMPs generated the highest inflammatory response in more than one recipient, indicating scarce specificity in the mucosal inflammatory reaction.

### Test Validation in UC Patients

We recruited 4 patients (3 men; median age, 54 years) with extensive moderate UC and refractory to several lines of immunosuppressive and biological therapies. The patients’ condition had lasted a median of 6.5 years (range, 4–27 years). **Table 1** summarizes the baseline characteristics of the 4 patients. Rectal biopsies from these patients yielded a low number of cells, limiting the compatibility test to 3 donor candidates.

**Table 1:** Clinical characteristics of the four patients with ulcerative colitis.

Patient	Age, sex	Extension^1^	Years from diagnosis	Treatment before FMT^2^	Steroid dose at FMT
UC1	42, male	E3	27	AZA, MCP,IFX, ADA, GOL, VDZ, TACRO	15 mg
UC2	41, male	E3	5	AZA, IFX,ADA,VDZ	none
UC3	71, female	E3	4	IFX,MTX, VDZ, ADA, TACRO	4 mg
UC4	66, male	E3	8	AZA,MCP,ADA,IFX, MTX, GOL, VDZ	10 mg

^1^Following Montreal classification.

^2^FMT, fecal microbiota transplantation; AZA, azathioprine; MCP, mercaptopurine; IFX, infliximab; ADA, adalimumab; GOL, golimumab; VDZ, vedolizumab; TACRO, tacrolimus; MTX, methotrexate.

**Figure 4:**
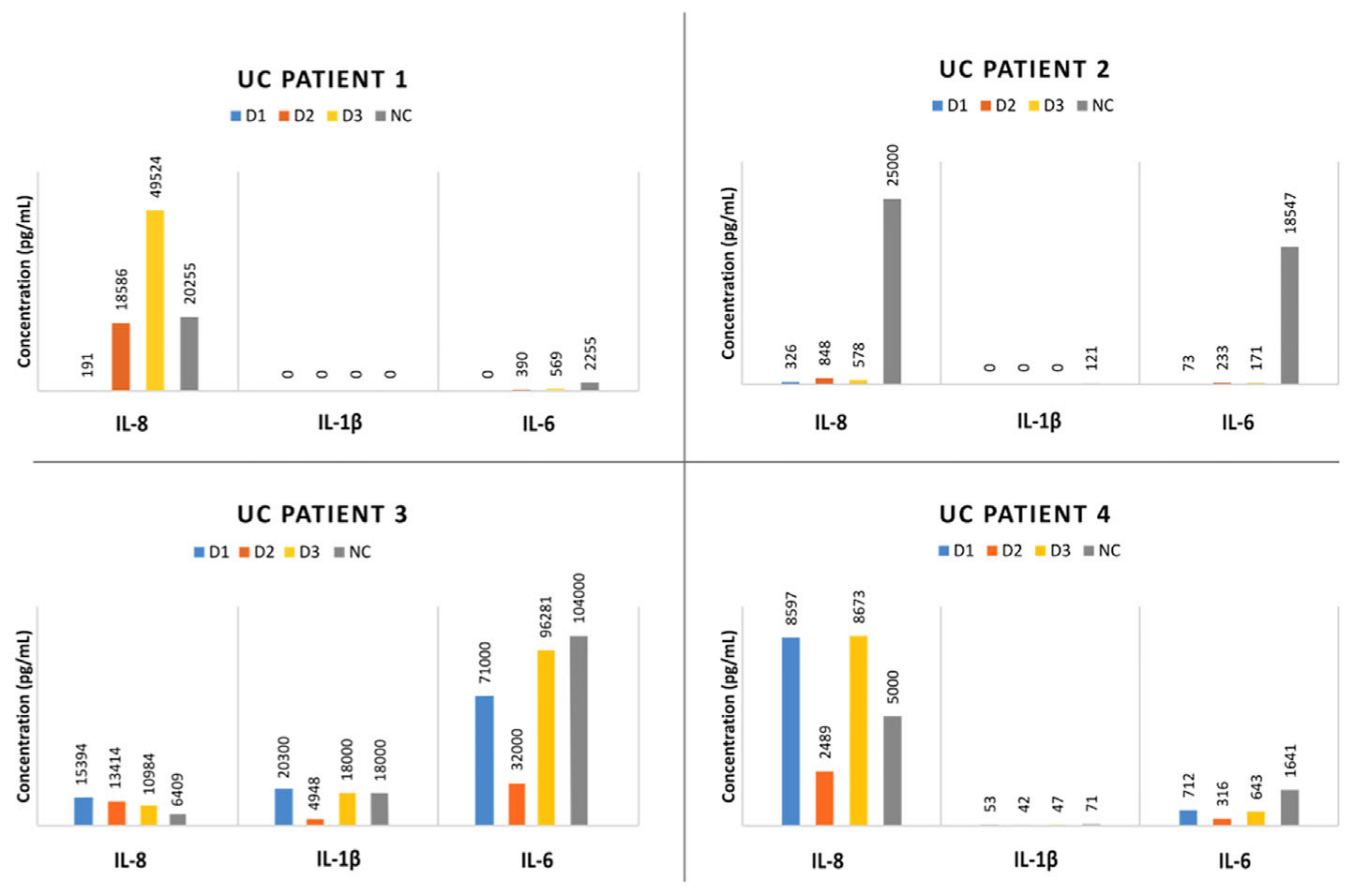
Results from *in vivo* evaluation of the test using 4 patients with ulcerative colitis (UC). Each quadrant showed the cytokine production of each patient against the 3 donor candidates’ microbiota. The basal cytokine production (without any antigenic stimulus) of the immunocompetent cells is also depicted (named as NC, negative control).

**Figure 4** shows the results of the compatibility test for each UC patient. Fecal donor candidate 1 was selected for patients 1 and 2, as this individual generated the mildest immune response in both cases, while donor candidate 2 was chosen as the FMT donor for patients 3 and 4. It should be noted that the baseline cytokine expression levels were much higher than those observed in the assays with cadavers, which might be due to the patients’ chronic inflammation state due to their colitis. Again, TNFα, IL-10, and IL-12 were not useful markers for donor selection, because they showed baseline or null expression levels.

We performed the FMT procedures with no noticeable adverse effects in any patient. First, a fecal infusion with 100 gr of fresh feces was performed by colonoscopy, followed by 4 rectal enemas obtained from another 100 g of stool and self-administered 1 per week with a syringe containing 50 ml, which was kept frozen at -20°C until use.

### FMT Engraftment in UC *vs* CDI

We performed 16s rDNA sequencing on the 22 fecal samples from the 4 patients with UC, the 2 selected donors, and the 2 patients with CDI, yielding 3,644,526 reads, whereas the negative control only yielded 123 reads, discarding external contamination. After quality filtering, chimera removal, merging, and discarding the very low-frequency sequences, we ultimately obtained a total of 1,739,846 reads. After the taxonomic assignment, nonbacterial sequences were removed, and 1,737,688 reads of 1402 ASVs were ultimately considered. For the diversity analysis, all samples were normalized *via* rarefaction, which was set to 23,405 sequences to maximize the retention of samples and to preserve representative diversity.

Alpha diversity measures the ecosystem’s degree of richness, to wit, the number of different species and their relative abundance. The lowest alpha diversity values were observed for the patients with CDI (3.8 ± 1.1 Shannon, 87.0 ± 28.2 Chao1, and 6.8 ± 0.1 Faith’s PD), followed by the patients with UC (5.0 ± 0.5 Shannon, 154.7 ± 46.4 Chao1, and 10.4 ± 2.0 Faith’s PD) and fecal donors (5.6 ± 0.6 Shannon, 286.0 ± 82.0 Chao1, and 16.4 ± 2.9 Faith’s PD). The alpha diversity metrics of the patients with UC were significantly increased (p<0.007) after FMT (287.7 ± 69.0 Chao1 and 15.9 ± 1.9 Faith’s PD), suggesting the incorporation of new bacterial species after the procedure. In contrast, the alpha diversity of the patients with CDI showed a slightly but not statistically significant increase (p>0.05) after FMT (4.3 ± 0.7 Shannon, 120.5 ± 37.5 Chao1, and 10.1 ± 2 Faith’s PD).

The fecal microbiome composition is detailed in the supplementary material (**Figure S2**). Up to 227 genera were detected in the whole population; the pre-FMT CDI samples had the lowest count (n=63), and the post-FMT UC samples had the highest count (n=206). The microbiome from the patients with CDI had considerable differences with regard to the rest of the samples, highlighting the abundance of the *Proteobacteria* phylum (29.8%), and *Streptococcus* (25.4%), *Ruminococcus* (14%), and *Weissella* (8.4%) genera. Conversely, the microbial composition of the donors and pre-FMT UC samples were more similar, highlighting the proportions of the *Bacteroidota* (15.7/12.3%) and *Verrucomicrobiota* (10.9/0.8%) phyla and the *Agathobacter* (10.6/8.1%) and *Akkermansia* (10.9/0.5%) genera. The most relevant differences between the donors and the pre-FMT patients with UC focused on *Firmicutes* (70.6/76.5%) and *Actinobacteriota* (2.5/7.7%) phyla and *Faecalibacterium* (6.7/10.9%) and *Bacteroides* (8.6/11.8%) genera.

The impact of FMT on the patients with UC resulted in the enrichment of *Bacteroidota* phylum (12.3/22.9%) and *Prevotella* genus (0.8/6.2%) and the decrease of the *Actinobacteriota* phylum (7.7/3.7%) and *Agathobacter* genus (8.1/3.0%). We should also mention the deficient engraftment of *Akkermansia* after FMT (0.8%), despite it being the most abundant genera in the donors (10.9%). In contrast, FMT in the patients with CDI produced a noticeable enrichment of the *Akkermansia* genus (0.1/17.7%) and a decrease of the *Firmicutes* phylum (63.7/45.8%) and *Ruminococcus* genus (14.0/0.9%).

**Figure 5:**
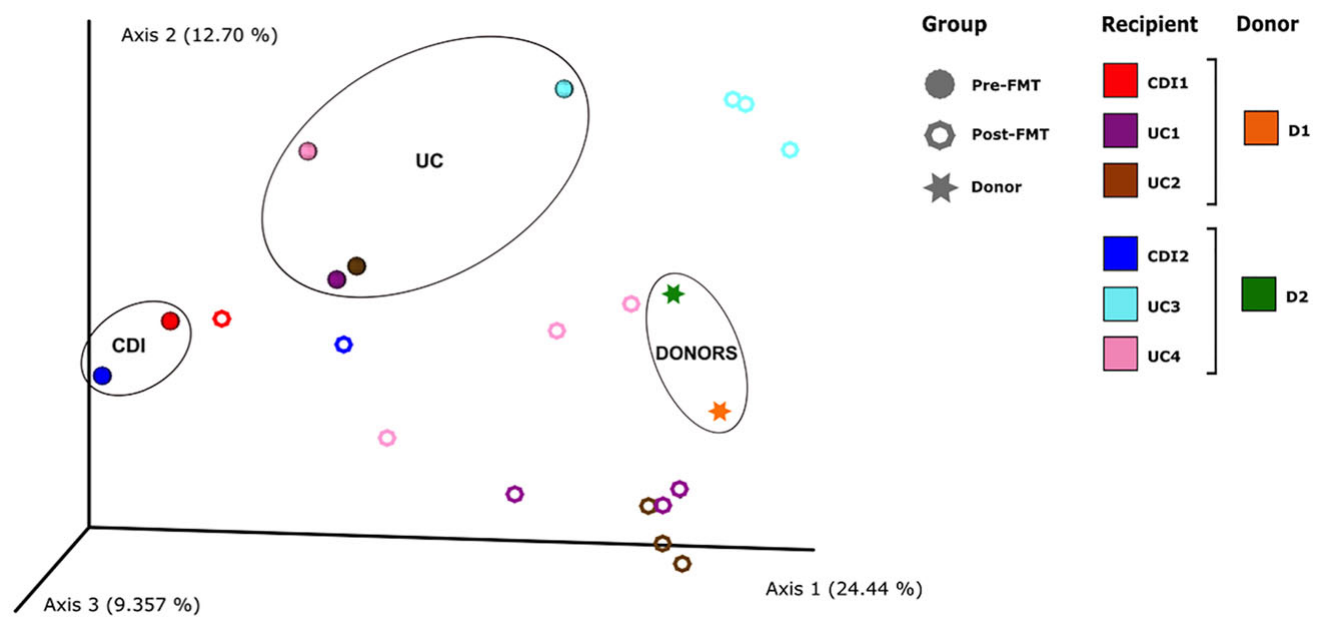
PCoA based on unweighted UniFrac distances calculated from the microbiological profiles of stool samples. Each point represents a sample, color indicates each subject, and the shape indicates if a sample was obtained before or after fecal microbiota transplantation (FMT), or whether it comes from a donor. In addition, brackets indicate which donor were selected for each patient.

Microbial communities can also be compared using beta diversity distances. We calculated the unweighted UniFrac distances between samples and used the distances to build a principal coordinates analysis plot (**Figure 5**). Although the analysis failed to detect statistically significant clusters, likely related to the small sample size, the visual inspection of the plot showed certain relevant trends. First, the donors, the patients with CDI, and those with UC could be separated by their microbiome composition; the patients with UC were the least homogeneous group. Second, FMT appears to have little effect on acquiring new taxa in the patients with CDI. Conversely, the patients with UC appear to be more affected by FMT through the acquisition of certain new taxa from donors to recipients (agreeing with the aforementioned increase in alpha diversity values after FMT), a situation that did not occur for UC patient 3, whose microbiome was barely modified by the intervention.

### Clinical Follow-Up of Patients With Ulcerative Colitis

**Figure 6:**
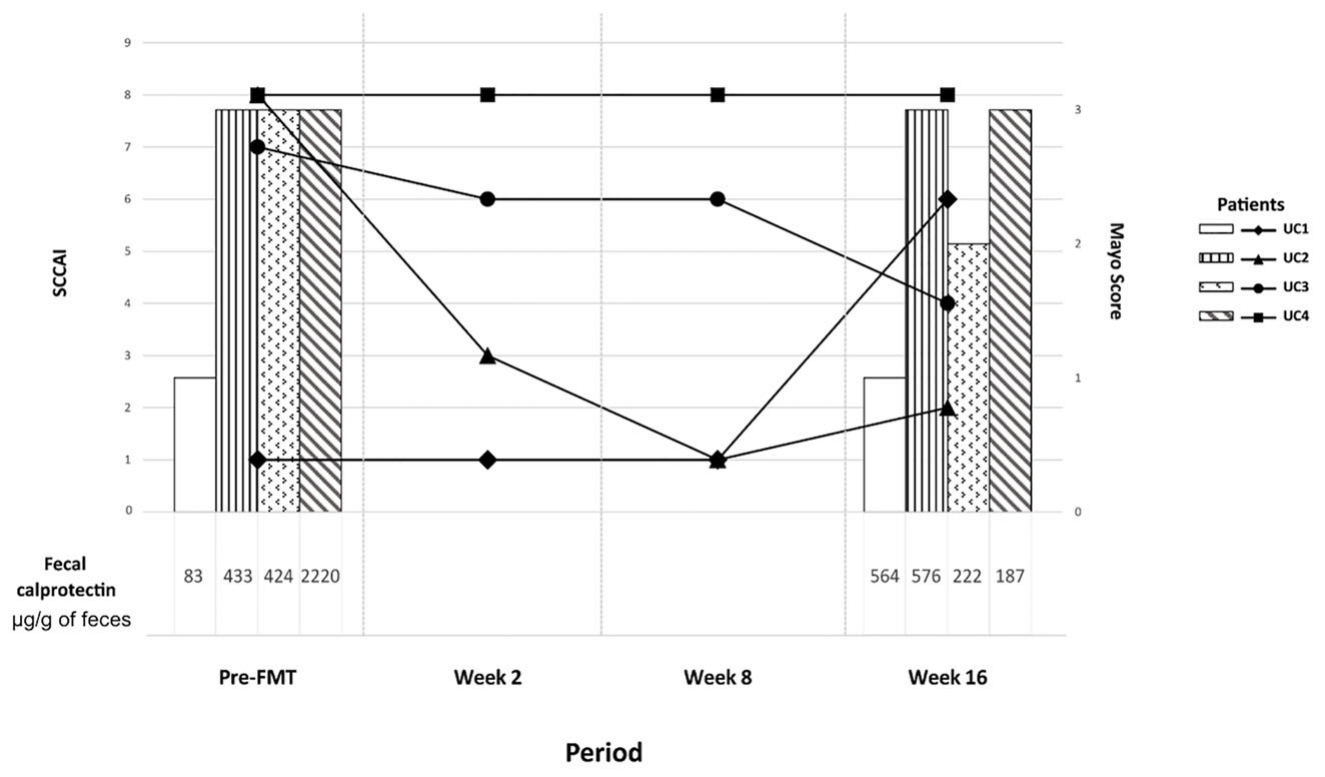
Clinical evolution of the four patients with ulcerative colitis over time, from the moment before fecal microbiota transplantation (FMT) (left) to the end of the follow-up at week 16 (right). The solid lines represent the SCCAI value at each time, whereas the bars (only recorded before FMT and at week 16 after FMT) showed the Mayo endoscopic score. Finally, fecal calprotectin levels for each patient before FMT and at week 16 were showed under the X axis.

**Figure 6** summarizes the results of the clinical follow-up of the patients with UC. The colonoscopy at 16 weeks showed no significant improvement in disease activity in any of the patients. Two patients (2 and 3) showed a slight decrease in SCCAI over time, whereas patient 1 had an increase in this value after FMT. Lastly, fecal calprotectin levels were comparable among the time points or even higher after the procedure (except for patient 4).

## Discussion

UC is an inflammatory bowel disease in which the dialogue between the gut microbiota and mucosal immunity is affected (2, 21, 22). FMT has been proposed as a useful tool for modifying the microbial gut ecosystem. Although FMT has a clinical efficacy in CDI of approximately 90%, it rarely achieves rates above 40-50% in UC (11, 23). Given that the immunological factor is particularly relevant in these patients, our present study proposed exploring the recipient’s immunological compatibility with respect to the antigenic MAMPs determinants of different donors to optimize the selection.

The first objective was to determine whether the inflammatory response of peripheral mononuclear cells could be comparable to those obtained from the gastrointestinal mucosal tract for use as a noninvasive sample. However, the results highlighted the relevance of the cells’ origin; while the inflammatory response of peripheral blood was overwhelming (as probably occurs during a bacteremia process), the jejunal mucosal cells exhibited a considerably lower reaction, close to tolerance. In fact, there was noticeably low inflammatory reaction against most of the tested MAMPs, which was also reproduced in rectal cells obtained from UC patients. This tendency could be related to frequent exposure to alien microorganisms that could be ingested with food or after environmental exposure.

One strength of the *in vitro* optimization of the test was the use of jejunal surgical specimens to obtain immunocompetent cells from the mucosa, which was motivated by: 1) the possibility of recovering a larger number of cells, and 2) the opportunity to reproduce the most functional model possible. Obviously, the colon is the natural target for FMT, especially when using the colonoscopy route; however, immune cells are mostly concentrated in the small intestine. The scheme we proposed allowed us to compare the intra-recipient reaction against 19 microbial ecosystems, and although the inflammatory status of these cadavers is not the most suitable, the results showed considerable variability, demonstrating individual compatibilities.

The first FMT studies on UC pointed to limited efficacy, although recent revisions (11, 23) observed higher remission when using high fecal dosage, regardless of the donor, and the previous administration of antibiotics; however, certain other variables might also be involved (24), as the duration and severity of the disease (11). None of our 4 patients showed relevant clinical improvement after FMT, which contrast with previous reports (25–27); however, the beneficial effects reported in those studies were not long lasting. Conversely, a recent study (28) provided evidence for the long-term efficacy of FMT in patients with UC, particularly associated with the increase in *Proteobacteria* and the decrease in *Bacteroidota* phyla. Other authors have suggested the role of the abundance of *Eggerthella, Lactobacillus*, and *Ruminococcus* before FMT or in the days immediately following the procedure as a predictive marker for maintaining clinical remission (29). It is important to note that our patients were particularly refractory to both immunosuppressive and biological therapies, which could negatively affect the clinical efficacy of FMT. In that sense, higher FMT success rates have been reported in young patients with UC with moderate activity compared with older patients or those with more aggressive clinical presentations (30), which is probably related to a higher retention of the bacterial dialogue with the mucosal immune system. Beyond the donor characteristics, the high genetic and microbiological variability of the recipient should also be taken into account in order to obtain FMT success (11).

Our results demonstrated relevant changes in microbiome composition provoked by FMT, with a greater impact on UC than CDI and including the acquisition of previously undetected bacterial taxa, although this was not associated with clinical improvement. The most noticeable result was the lack of *Akkermansia* intestinal engraftment in the patients with UC, whereas the abundance of this genus in the patients with CDI after FMT was comparable with that observed in the donors. These observations exemplify how the intestinal tract lumen, which strictly should not be considered part of our body, harbors a resident microbiota that competes with the transferred ecosystem after FMT, limiting colonization. This battle appears to depend more on bacterial fitness that on the immune reaction, given the tolerant response observed in most tests. Consequently, immunological compatibility is not an essential factor to explore when selecting stool donors for FMT, although, in a subsequent stage, we should analyze the interactions between the two microbiota to select the donor most likely to displace the recipient’s resident microbiota.

The limitations of this study are the low number of patients with UC recruited for FMT and their clinical characteristics of long-term refractory disease. In addition, the use of more selective and specific immunological markers, such as those related to the inflammatory TH-17/TH-1 axis (31) or Treg pathways (32) might be more appropriate or discriminating; however, the success of FMT does not appear to depend on immunological factors. To obtain mucosal-associated immune cells in the patients with UC, we employed rectal biopsies; however, the numbers of recovered cells from these samples were limited and conditioned the assay to only 3 donor candidates, and results obtained with jejunal cells are not completely reproducible at this location. Finally, achieving MAMPs by heat inactivation could not be the best approximation, but co-culture was ruled out because of cell apoptosis due to bacterial growth.

In summary, we designed, optimized, and evaluated a compatibility test for FMT donor selection based on the inflammatory reaction of mucosal immune cells against fecal microbiota, although we cannot to rule out other immune response pathways non-explored in our work and that could be critical in the recipient compatibility with the microbiota of different donors. Our experiments with immune cells obtained from the jejunal mucosa of organ donors and from rectal biopsies of patients with UC showed that tolerance with scarce or null inflammatory activation was the most frequent finding, ruling out the requirement of our test. Microbial replacement by FMT was demonstrated in both the CDI and UC models using shared donors. Although we suggest that clinical success in UC could be more dependent on the microbial interactions than on the immunological influence, the higher efficacy for CDI could be a consequence of other unexplored factors.

## Data Availability Statement

The original contributions presented in the study are publicly available. This data can be found at https://www.ncbi.nlm.nih.gov/Traces/study/?acc=PRJNA702025&o=acc_s%3Aa.

## Ethics Statement

The studies involving human participants were reviewed and approved by CEIC Hospital Ramón y Cajal, Madrid, Spain. The patients/participants provided their written informed consent to participate in this study.

## Author Contributions

MP-A, CG-H, RC, GR, and RC conceived the experiments and supervised the final manuscript. JN, PN, AM-P, JO, and JG-P provided the surgical pieces. AG and AL-S recruited and clinically followed the patients with UC. All authors contributed to the article and approved the submitted version.

## Funding

This work was supported by the Instituto de Salud Carlos III, PI17/00115 and PI20/00164 to RdC, and REIPI (RD16/0016/0011) actions, cofinanced by the European Development Regional Fund “A way to achieve Europe” (ERDF). Also, MPA was supported by a Rio Hortega contract (CM19/00069) from the Instituto de Salud Carlos III and the European Regional Development Fund (ERDF, “A way to achieve Europe”).

## Conflict of Interest

The authors declare that the research was conducted in the absence of any commercial or financial relationships that could be construed as a potential conflict of interest.
