# Supplementary material for: An Immunologic Compatibility Testing Was Not Useful for Donor Selection in Fecal Microbiota Transplantation for Ulcerative Colitis
Source: Front Immunol. 2021 Jun 4;12:683387. doi: 10.3389/fimmu.2021.683387 (PMC8212046; doi:10.3389/fimmu.2021.683387)
Supplement: Supplementary file 1 [file Table_1.docx]

**SUPPLEMENTARY DATA**

**Table S1.** Faecal samples which were collected and submitted to 16S rDNA massive sequencing. Raw sequencing data are publicly available through accession numbers.

| NCBI accession number | Sample name | Subject^1^ | Collection time^2^ |
| --- | --- | --- | --- |
| SAMN17922164 | UC1-pre | UC1 | pre-FMT |
| SAMN17922165 | UC1-15d | UC1 | 15 days after FMT |
| SAMN17922166 | UC1-30d | UC1 | 30 days after FMT |
| SAMN17922167 | UC1-60d | UC1 | 60 days after FMT |
| SAMN17922168 | UC2-pre | UC2 | pre-FMT |
| SAMN17922169 | UC2-15d | UC2 | 15 days after FMT |
| SAMN17922170 | UC2-30d | UC2 | 30 days after FMT |
| SAMN17922171 | UC2-60d | UC2 | 60 days after FMT |
| SAMN17922172 | UC3-pre | UC3 | pre-FMT |
| SAMN17922173 | UC3-15d | UC3 | 15 days after FMT |
| SAMN17922174 | UC3-30d | UC3 | 30 days after FMT |
| SAMN17922175 | UC3-60d | UC3 | 60 days after FMT |
| SAMN17922176 | UC4-pre | UC4 | pre-FMT |
| SAMN17922177 | UC4-15d | UC4 | 15 days after FMT |
| SAMN17922178 | UC4-30d | UC4 | 30 days after FMT |
| SAMN17922179 | UC4-60d | UC4 | 60 days after FMT |
| SAMN17922180 | CDI1-pre | CDI1 | pre-FMT |
| SAMN17922181 | CDI1-60d | CDI1 | 60 days after FMT |
| SAMN17922182 | CDI2-pre | CDI2 | pre-FMT |
| SAMN17922183 | CDI2-30d | CDI2 | 30 days after FMT |
| SAMN17922184 | D1 | Donor 1 | - |
| SAMN17922185 | D2 | Donor 2 | - |

1: UC (ulcerative colitis), CDI (*Clostridioides difficile* infection)

2: FMT (fecal microbiota transplantation)
