# Supplementary figures and images for: An Immunologic Compatibility Testing Was Not Useful for Donor Selection in Fecal Microbiota Transplantation for Ulcerative Colitis
Source: Front Immunol. 2021 Jun 4;12:683387. doi: 10.3389/fimmu.2021.683387 (PMC8212046; doi:10.3389/fimmu.2021.683387)

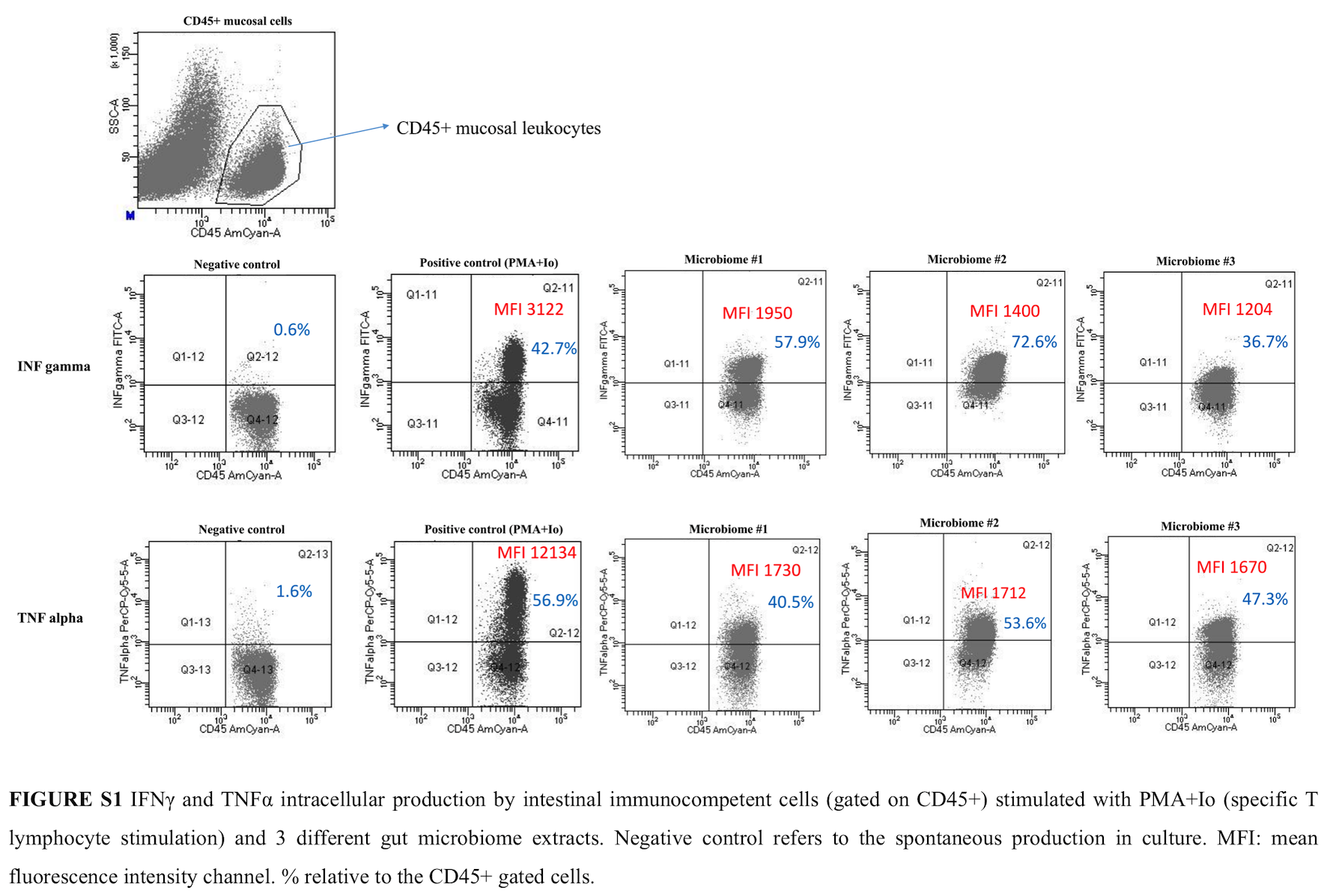

Supplement: Supplementary file 2 [file Image_1.tiff]

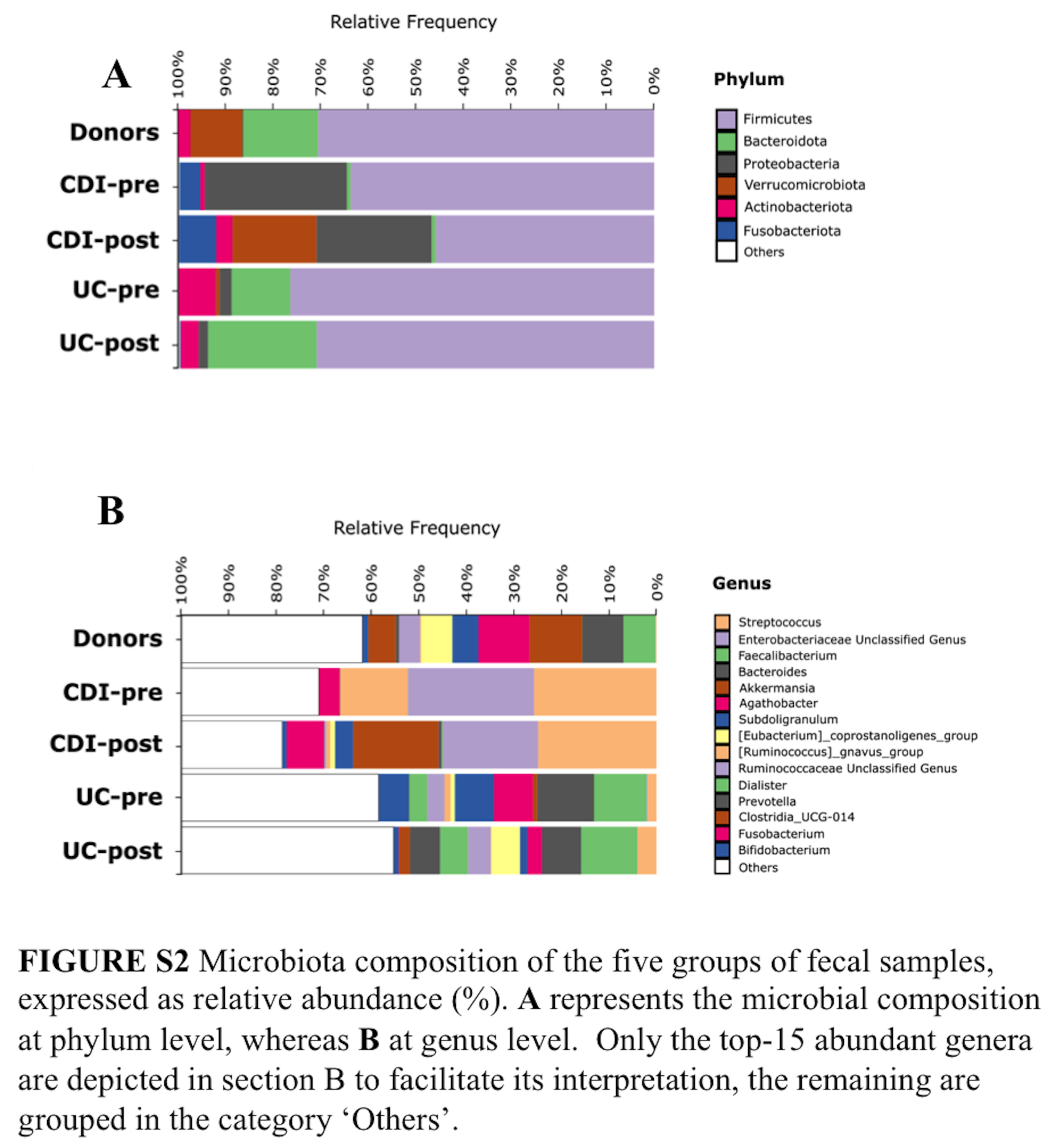

Supplement: Supplementary file 3 [file Image_2.tiff]
